# Characterization of Decellularized Extracellular Matrix from Milkfish (*Chanos chanos*) Skin

**DOI:** 10.3390/biomimetics7040213

**Published:** 2022-11-24

**Authors:** Ronald Bual, Marionilo Labares, Kit Dominick Don Valle, Job Pague, Zesreal Cain Bantilan, Princess Grace Ducao, Johnel Alimasag, Catherine Acibar

**Affiliations:** 1 Department of Chemical Engineering and Technology, College of Engineering and Technology, Mindanao State University-Iligan Institute of Technology, Iligan City 9200, Philippines; 2 Center for Sustainable Polymers (CSP), Mindanao State University-Iligan Institute of Technology, Iligan City 9200, Philippines

**Keywords:** milkfish, fish skin, decellularization, wound healing, extracellular matrix, collagen, fish processing wastes, acellular dermal matrix

## Abstract

Milkfish (*Chanos chanos*) is an abundant fish commodity in the Philippines that generates a large number of wastes such as skin, scales, viscera, and bones, which, upon disposal, cause environmental pollution. The abundance of these wastes, such as fish skin, rich in bioactive natural products such as collagen, elicits interest in their conversion into high-market-value products. The decellularization of milkfish skin waste can extract its extracellular matrix (ECM), a potential raw material for biomedical applications such as the repair of damaged skin tissues. In particular, this study characterized the developed decellularized ECM with different concentrations (0.1%, 1.0%) of the decellularizing agents (Triton X-100, SDS) and temperature (4 °C, room temperature) using milkfish skin. The decellularized ECM structure was better preserved using Triton X-100, while SDS was more effective in cell component removal, especially at 1% concentration and 4 °C temperature. There were significant effects of varying the temperatures and concentrations on the physical and mechanical properties of the decellularized ECM. Future studies could explore more variables to further establish protocols and more analyses to better characterize the decellularized milkfish skin.

## 1. Introduction

Milkfish *(Chanos chanos)* is a saltwater fish characterized by silver color cultured in shallow coastal water areas and is capable of living in low-salinity water [1]. Being labeled as the leading cultured fish in the Philippines [2], a large number of wastes are being generated, such as skin, scales, and bones, that cause environmental pollution when disposed of. The annual increase in milkfish production, according to the Philippines’ Department of Agriculture, has increased to 3.74% in 2019 alone [3]. This elicits interest in converting these wastes into high-market-value products, especially milkfish skin as a significant protein source [4].

The milkfish skin is rich in bioactive natural products and is approximately 50% collagen [5]. This can be used as a raw material for biomedical applications such as the repair of damaged skin tissues caused by burns. A promising treatment for these injuries is the use of acellular dermal matrix (ADM) derived from the collagen-rich extracellular matrix (ECM) of milkfish skin. This would provide a structural matrix that would allow for dermal and epidermal regeneration [6]. Additionally, the absence of prions or viruses that can be transmitted to humans and high antimicrobial properties [7] also make the fish skin advantageous for this type of biomedical application. Correspondingly, to achieve a functional ECM from milkfish skin, the process called decellularization is used.

Decellularization is the process of removing the cellular compartment of living tissues, creating an acellular ECM scaffold that can subsequently be used for varying purposes [8]. The removal of cellular compartments such as allogeneic or xenogeneic cellular antigens would prevent immune response and leave only an intact ECM [9]. Decellularized ECM is obtained by a defined sequence of physical, chemical, and enzymatic treatments [10]. However, there is a challenge in how maintaining the structure and function of the complex mixture of proteins that make up the ECM [7,11]. Several studies have already been published on the use of the detergents Triton X-100 (TX100) and sodium dodecyl sulfate (SDS) in the production of decellularized ECM [10]. A previous study reported that among the decellularization agents used in their study, TX100 produced the best ECM in terms of structure, toxicity, cell adhesion, and mechanical and physical properties [7]. On the other hand, SDS is already known to be an essential reagent for the decellularization of numerous organs [12].

Although detergents are effective in removing cellular components, it is also known that they have a cytotoxic effect on the cell [13,14]. Aside from the removal of lysed cellular material, the effectivity of decellularized extracellular matrix also depends on the removal of cytotoxic detergents after decellularization, especially SDS, which is known to be difficult to remove [14,15].

The goal of this research is to determine how varying the temperature and concentration of decellularizing agents will affect the removal of cellular components from milkfish skin while preserving the ECM. The physical, mechanical, and thermal characteristics of the developed decellularized ECM and residual detergent after decellularization were also examined.

## 2. Materials and Methods

The sample preparation and decellularization were carried out by modifying the methods used by White et al. [16] and Oliveira et al. [17].

### 2.1. Preparation and Decellularization of Milkfish Skin

Milkfish that were around 300–500 g were obtained from a local fish market. The skin was obtained after the descaling of the fish. The skin was washed with distilled water and then cut into 1 *×* 1 in^2^ and 1 *×* 3 in^2^ sections. The skin sections were pre-washed with phosphate-buffered saline (PBS) solution for 15 min to remove any impurities and preserve the samples.

PBS-washed skin sections were decellularized using TX100 (Loba Chemie, Mumbai, India) and SDS (Loba Chemie, Mumbai, India). To determine the effects of varying the concentration of decellularizing agents and temperature, eight protocols were performed to produce the decellularized ECM, as shown in Table 1 below.

The skin sections were soaked in different decellularizing agents of varying concentrations for 24 h with agitation using magnetic stirrers. The decellularization was carried out at 25 °C at room temperature while the rest of the samples were carried out inside 4 °C refrigerators.

After 24 h, the samples were immediately washed thrice with PBS for 15 min at constant agitation. The decellularization was continued for another 24 h using freshly prepared agents, then rewashed again thrice with PBS. All agitations were performed using magnetic stirrer at 300 rpm.

The decellularized skin was stored at −80 °C in an ultralow-temperature refrigerator (Haier, Qiangdao, China) for at least 24 h prior to lyophilization using a freeze dryer (Gyrozen, Gimpo, Republic of Korea) at −55 °C for another 24 h.

### 2.2. Histological Staining

Around 4 × 4 mm^2^ of the samples were fixed using buffered 10% formaldehyde for 36 h. The fixed samples were then embedded and blocked using paraffin wax [7]. The paraffin blocks were cut using a microtome to obtain 4 µm-thick ribbons [18]. The samples were then mounted on glass slides and stained with hematoxylin and eosin (H&E) solution. Hematoxylin stains the acid structures, such as DNA and nuclei, in blue-purple, while eosin stains the basic cell components, such as proteins, in pink. The stained samples were studied under a CX22 laboratory microscope (Olympus, Tokyo, Japan) to examine the decellularized tissues.

### 2.3. DNA Extraction and Quantification

DNA extraction was performed using DNeasy Blood & Tissue Kit (Qiagen^®^, Valencia, CA, USA), while the quantification of DNA presence was conducted using Qubit™ 1X dsDNA HS Assay Kits (Thermo Fisher Scientific, Waltham, MA, USA). The tests were conducted in accordance with the manufacturer’s user manuals.

### 2.4. Composition Analysis Using Attenuated Total Reflectance—Fourier Transform Infrared (ATR–FTIR) Spectroscopy

The sample was placed on the spectrum plate of the QATR-10 single reflection FTIR (Shimadzu, Kyoto, Japan). Infrared rays with a wavelength range from 400 to 4000 cm^−1^ were allowed to pass through the spectrum plate. The amount of absorption of wavelength is determined by the automated software and visualized through the ATR–FTIR spectrum as a percentage of infrared wavelength transmittance. If preserved, the bonds associated with collagen in the decellularized ECM were detected on the ATR–FTIR spectra.

### 2.5. Physical and Mechanical Properties

#### 2.5.1. Tensile Strength

The mechanical strength was determined by uniaxial tensile testing following ASTM D882 with modifications. The decellularized 1 × 3 in^2^ skin sections were cut into dumbbell-shaped stencils using a die cutter (L = 50.81, W = 2.25 mm) and then secured into the Autograph AGS-X Universal Tester (Shimadzu, Kyoto, Japan). The testing was performed using a 500N load cell and an extension rate of 10 mm/min. The maximum tensile stress was determined from the software Trapezium X of the universal tester.

#### 2.5.2. Hydrophilicity

The surface hydrophilicity, or the ability of a material to attract water [19], was determined by measuring the contact angle formed by a drop of water on the sample’s surface. The sample on a glass slide was placed on the stage of Theta Lite Optical Tensiometer (Biolin Scientific, Stockholm, Sweden) then a drop of water (approximately 1–2 µL) was introduced on the sample’s surface. The sample was considered hydrophilic when the water contact angle was <90°; otherwise, it was hydrophobic when the angle was >90° [20]. Both sides of the skin sample were tested for hydrophilicity.

#### 2.5.3. Water Absorption

Water absorption, or water uptake capacity of the samples, was performed by slightly modifying the procedures in ASTM D570-98 and ASTM C20-00. The samples were dried in an oven for 2 h at 50 °C and then weighed after cooling in a desiccator. The dried samples were soaked in a 23 °C distilled water for 2 h and then blotted using a cotton cloth to remove the drops of water from the surface before weighing. The water absorption expressed in percentage was calculated as follows:

% Water Absorption = [(Wet Weight − Dry Weight)/Dry Weight] × 100.(1)

### 2.6. Thermal Degradation and Denaturation Profile

#### 2.6.1. Thermogravimetric Analysis (TGA)

The thermal degradation of the samples was analyzed through the thermogravimetric method using DTG-60H (Shimadzu, Kyoto, Japan). An initial sample weight of approximately 30 mg was analyzed and subjected to temperatures from 30 °C to 700 °C with a heating rate of 10 °C/min [21]. The sample weight change and the temperature of the oven were recorded.

#### 2.6.2. Differential Scanning Calorimetry (DSC)

The denaturation temperature was determined by analyzing small cuts of the sample weighing approximately 5 mg on a DSC 4000 (Perkin Elmer, Waltham, MA, USA). The heating rate was set to 10 °C/min, and the temperature range of 30 °C to 300 °C.

### 2.7. Residual Detergents Determination

The methods to determine the concentration of residual detergents in the decellularized ECM were performed using UV-VIS Spectrophotometry. For residual Triton X-100, the method performed by Pavlović et al. was followed with modifications on the concentration of standards used [22]. For residual SDS, the methylene blue (MB) examination performed by Alizadeh et al. was followed [14].

### 2.8. Statistical Analysis

The quantitative data presented were all reported as mean ± standard deviation of the mean and were analyzed using a one-way analysis of variance (ANOVA) test, followed by Tukey’s multiple comparison test. There were statistically significant differences between the reported means when *p* < 0.05.

## 3. Results

### 3.1. Visual Assessment

**Figure 1:**
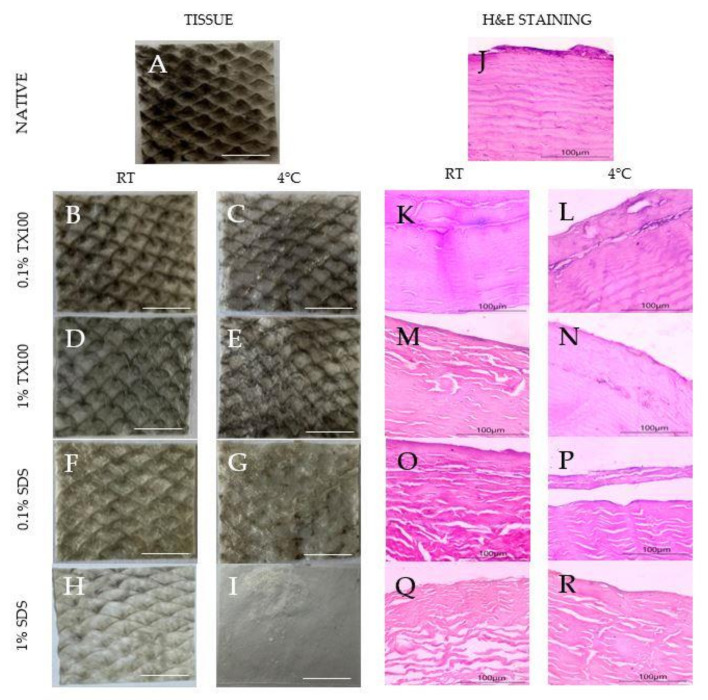
Images of the native and decellularized milkfish skin: (**A**–**I**) visual comparison for pigment removal (1× magnification), scale bar = 1 cm; (**J**–**R**) microscope images of hematoxylin and eosin-stained samples (10× magnification), scale bar = 100 μm.

The samples appeared to increase transparency because of the loss of pigments after decellularization, as shown in Figure 1A–I. The observation was evident, especially for samples that used decellularizing agents of higher concentrations. Moreover, samples that were decellularized at 4 °C appeared to be whiter than the samples carried out at room temperature. In addition, SDS appeared to be visually better at pigment removal than TX100.

### 3.2. Histological Staining

The hematoxylin and eosin (H&E) staining of the native and decellularized ECM is also shown in Figure 1J–R. The reduction in the blue-purple stains on the decellularized samples (Figure 1K–R) suggests the varying extent of cell component removal depending on the condition. Specifically, decellularization that was conducted at 4 °C showed higher cell component removal (Figure 1L,N,P,R). Structurally, decellularization using TX100 (Figure 1K–N) resulted in decellularized ECM that was more similar to the native sample regardless of the temperature. Contrarily, the use of SDS led to clear structural damage to the collagen fibers despite being more effective in cell component removal, especially at the higher concentration (Figure 1R).

### 3.3. DNA Extraction and Quantification

**Figure 2:**
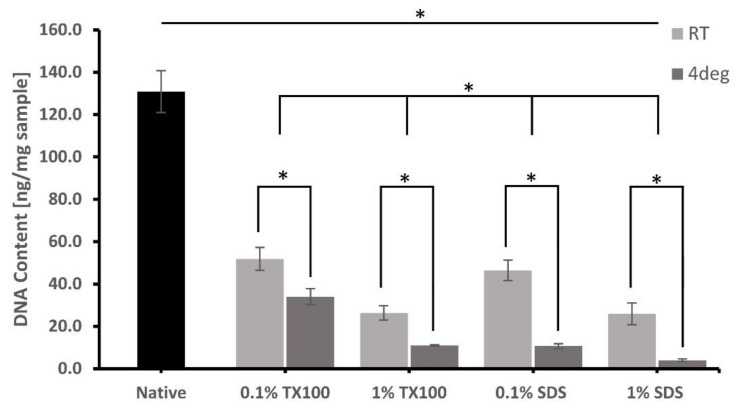
Comparative DNA quantification (ng/mg of the dry sample) of native milkfish skin and decellularized ECM developed from the different conditions. Bars represent standard deviation (*n* = 3). * *p* < 0.05.

The extent of cell removal after the decellularization was confirmed on the residual DNA of the ECM, as shown in Figure 2. Most of the protocols that were performed at 4 °C and used 1% concentration had significant DNA removal compared with the samples decellularized using 0.1% concentration at room temperature. Decellularized ECM developed using 1% SDS and decellularized at 4 °C has the lowest residual DNA of only 3.9 ± 0.65 ng/mg. Except only for 0.1% SDS at room temperature, all the residual DNA content of the decellularized ECM was less than 50 ng/mg, which is the level that can no longer cause immune stimulation and inflammation.

### 3.4. Composition Analysis Using Attenuated Total Reflectance—Fourier Transform Infrared (ATR–FTIR) Spectroscopy

**Figure 3:**
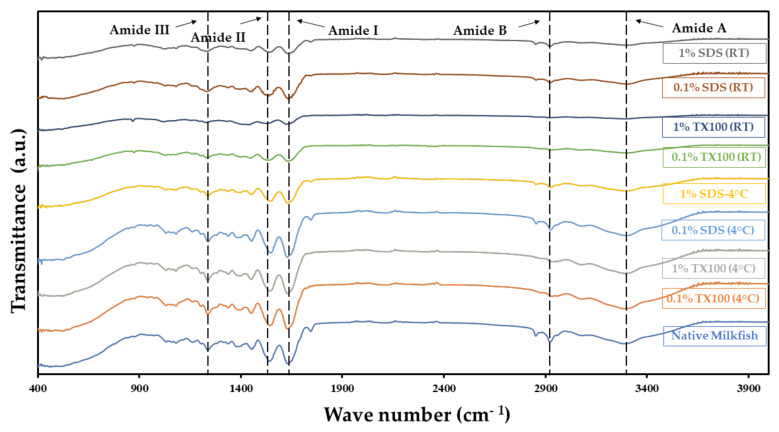
ATR-FTIR spectra of the native and decellularized milkfish skin show that amide bands were preserved after decellularization.

One of the most significant criteria of decellularization is the preservation of crucial ECM components and the intactness of structure. The ATR–FTIR spectra for the native and decellularized samples in Figure 3 showed relatively similar peaks and bands for different functional groups. This indicates that the decellularization process did preserve the main ECM component, collagen. The typical amide bands observed were the amide A band in the 3272–3240 cm^−1^ region, amide B in the 2940–3080 cm^−1^ region, amide I between 1700 and 1600 cm^−1^, amide II around 1532–1555 cm^−1^, and amide III around 1240 cm^−1^. The described bands agree with the literature [23], confirming that the decellularization process did not alter the composition of the ECM. The detected amides I, II, and III bands related to collagen components suggest that the ECM was preserved regardless of the conditions used in the study.

### 3.5. Physical and Mechanical Properties

#### 3.5.1. Tensile Strength

**Figure 4:**
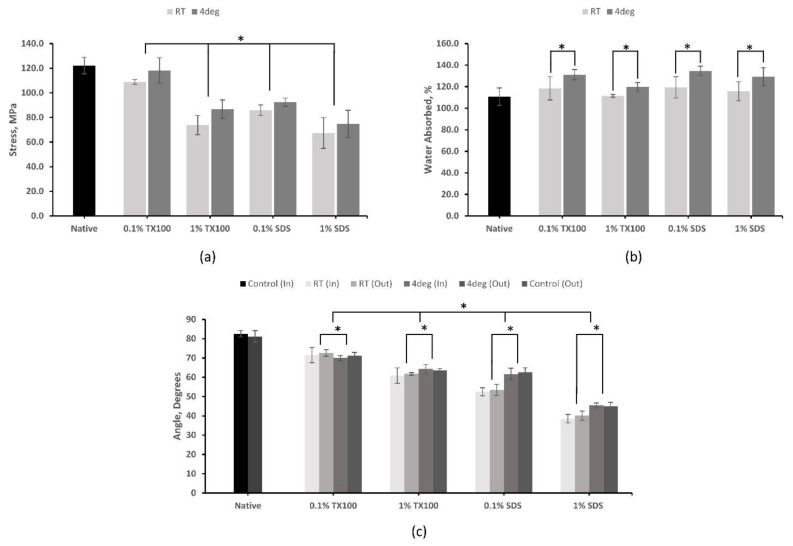
Physical and mechanical tests of native and decellularized milkfish skin: (**a**) tensile strength; (**b**) water absorption test; (**c**) hydrophilicity test measured using water contact angle. Bars represent standard deviation (*n* = 3). * *p* < 0.05.

The analysis using UTM, as shown in Figure 4a, has revealed that decellularization has negatively affected the tensile strength of the skin, as the removal of ECM components resulted in the deterioration of the mechanical properties of the tissue. The tensile strength of the decellularized ECM produced at 4 °C was higher compared with their room temperature counterparts. Decellularized ECM using 0.1% TX100 at 4 °C (118.14 ± 10.27 MPa) and 1% SDS at room temperature (67.22 ± 12.47) have the highest and lowest value for tensile strength, respectively. Samples treated with TX100, which can sufficiently preserve ECM, have higher tensile strength properties compared to that of SDS, which can disrupt collagen networks. Increased concentration resulted in coarser cell removal, which consequently decreased the tensile strength of the samples. Furthermore, statistical analysis indicated that temperature, decellularizing agent, and concentration have a significant effect on the tensile strength of the samples (*p* < 0.05).

#### 3.5.2. Hydrophilicity

The results of the contact angle test (Figure 4c) have shown that native skin has a hydrophilic property, and this property was enhanced in the decellularized ECM. Moreover, the decellularization process that consequently changes the surface condition resulted in a lower contact angle or increased hydrophilicity of the samples. The decellularized ECM developed using SDS had shown the highest hydrophilicity, especially when the concentration was 1.0%. This is an ideal property for ADM since more cells adhere to a hydrophilic material to facilitate cell attachment. Both sides of the skin were tested for hydrophilicity, and the results showed no significant difference (*p* = 0.51) in the contact angle. The increase in concentration resulted in a decrease in contact angle. However, there were significant effects on the contact angle in varying the concentration of the decellularizing agents and temperature (*p* < 0.05).

#### 3.5.3. Water Absorption

The decellularized samples have an increased ability to absorb water compared with the native sample, as shown in Figure 4b. Among the samples, the highest average swelling ratio was exhibited by the decellularized ECM using 0.1% SDS at 4 °C (134.66 ± 4.47%). Apparently, the decellularizations performed at 4 °C have higher water absorptivity compared with those protocols performed at room temperature. The lower concentration protocols also have higher water absorptivity. Furthermore, statistical analysis indicated that temperature and concentration do have a significant effect on the samples (*p* < 0.05). However, no significant difference was observed between the two decellularizing agents (*p* = 0.15). An ideal skin scaffold should absorb wound exudate and maintain the wound moist for a faster and more effective healing process.

### 3.6. Thermal Degradation and Denaturation Profile

#### 3.6.1. Thermogravimetric Analysis (TGA)

**Figure 5:**
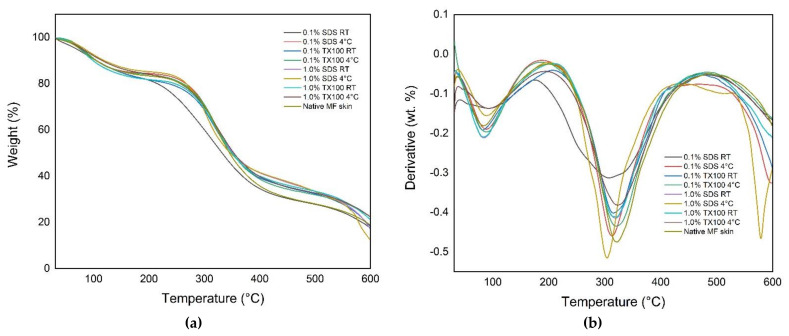
Thermal gravimetric curves for native and decellularized ECM: (**a**) %weight loss; (**b**) derivative of the %weight loss.

Thermogravimetric analysis (TGA) is useful for determining the thermal stability of the decellularized ECM. The thermal curves in Figure 5 show the characteristic temperatures and the mass-loss steps of the native and decellularized ECM. It was observed that there were two-step weight loss peaks on the curve for all the samples. Specifically, the decellularized ECM samples have shown that the first step or the initial degradation started from 30 °C to 205 °C. The second degradation step occurred at the temperature range from 190 °C to 490 °C. The samples that were decellularized at 4 °C had higher weight loss on the second degradation step compared with those samples treated at room temperature. In addition, the use of decellularizing agents with higher concentrations resulted in lower weight loss on the second degradation step compared with samples treated with lower concentrations.

#### 3.6.2. Differential Scanning Calorimetry (DSC)

**Figure 6:**
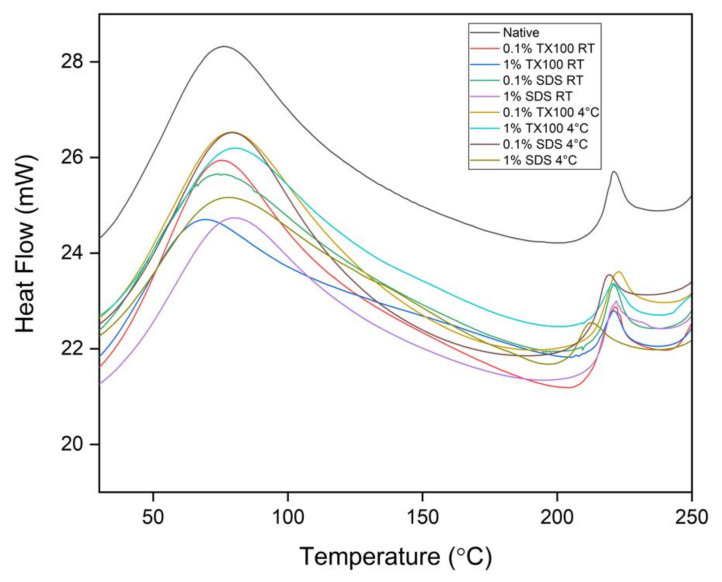
Differential Scanning Calorimetry (DSC) analysis of the native and decellularized ECM from milkfish skin.

The DSC analysis of all the samples revealed a single endothermal peak in the temperature range from 0 to 100 °C, as shown in Figure 6. This endothermal peak is associated with the thermal denaturation temperature of collagen and the release of free and bound moisture. Technically, the decellularization treatment has increased the thermal denaturation of the samples as compared to the native sample. It was also observed that the decellularized ECM developed at 4 °C have higher thermal denaturation temperature compared with those at room temperature. In addition, the higher concentration and the use of TX100 also resulted in higher thermal denaturation temperatures.

The second endothermal peaks were observed at around 213 to 223 °C. These peaks were related to the release of structural moisture, which is responsible for the stability of the triple helix structure of collagen. In general, the measured denaturation temperatures suggested that the decellularized ECM could withstand body temperature experiencing inflammation which is at about 42 °C.

### 3.7. Residual Detergent Determination

A higher concentration of residual detergent in the decellularized ECM was observed for both 1.0% TX100 and 1.0% SDS (*p* < 0.05) since more detergent must be removed during washing. The results also showed that there were lesser residual detergents when the decellularization was conducted at room temperature than at 4C (*p* < 0.05). In addition, protocols treated with SDS have higher residual detergent compared with using TX100 (*p* < 0.05). This is aligned with the findings of several previous studies on the difficulty of removing SDS compared with other anionic detergents such as TX100 [13,14].

**Figure 7:**
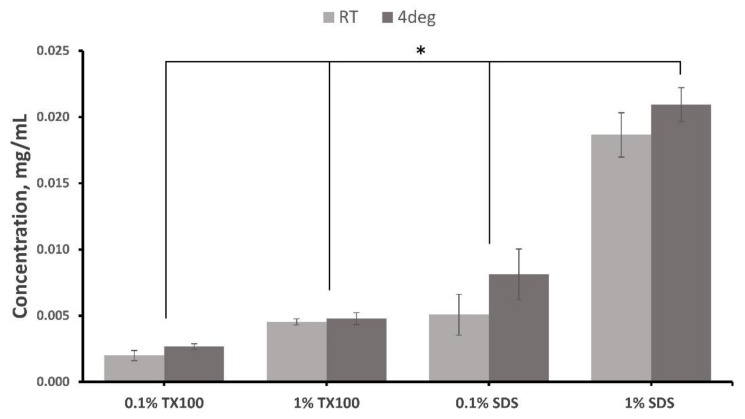
Residual detergent test for Triton X-100 and SDS in the decellularized extracellular matrix of milkfish skin. Bars represent standard deviation (*n* = 3). * *p* < 0.05.

There were no significant effects on the residual concentration of the detergent in the tissue between the interaction of the three variables (*p* = 0.086). The results of the residual detergent test of the decellularized ECM are shown in Figure 7.

## 4. Discussion

The conversion of fish wastes, such as milkfish skin, into high-value products, has environmental and economic advantages. Milkfish skin, rich in collagen [5], is a very viable material for skin tissue regeneration treatments. This is because fish skin exhibits similar properties to human skin in structural and mechanical tests of regenerative cell compatibility [7]. Unfortunately, there were no studies established yet for the decellularization specific to milkfish skin.

This paper illustrated the decellularization of milkfish skin using different decellularizing agents with varying concentrations and processed at different temperatures. Specifically, the process used sodium dodecyl sulfate (SDS) and Triton X-100 (TX100) as decellularizing agents and varied the concentration to 0.1% and 1.0%. The condition of the decellularization was also varied by conducting the treatment at 4 °C and room temperature. Hematoxylin and eosin staining revealed that TX100 at lower concentration tends to preserve the collagen fiber and the structural integrity of the ECM regardless of treatment temperature. On the other hand, SDS has a higher structural damaging effect which is magnified at higher concentrations but is more effective in cell component removal. These observations are consistent with other published studies [17,24,25,26].

Cell removal, which is evident in the absence of blue stains, was achieved more with decellularization using higher concentration and carried out at 4 °C. This was confirmed with the DNA quantification with maximum cell removal of 94.13% for TX100 and 98.21% for SDS, both at 1% concentration and temperature of 4 °C. The decellularized ECM was able to achieve the minimum residual DNA criteria of 50 ng DNA/mg sample to avoid adverse cell and host responses [7,24].

According to the ATR–FTIR spectra, it appears that more intense bands were seen at 4 °C compared with RT conditions. This finding might point to an unfavorable effect, such as the denaturation of ECM proteins. On the other hand, the tensile strength of the samples was negatively affected by decellularization. The decrease in tensile strength of the samples after decellularization was due to the removal of ECM components that deteriorates the mechanical properties of the tissues [27]. The structure-damaging effect of SDS, especially at higher concentrations, yielded the lowest tensile strength compared with TX100 treated samples. The use of SDS compromised the mechanical strength by seriously disturbing and disrupting collagen network structure [28,29]. On the other hand, TX100-treated tissues sufficiently maintained key ECM proteins and mechanical properties [26].

The decellularization has generally increased the hydrophilicity and water absorption ability of milkfish skin. The 1% SDS decellularized ECM at 4 °C was the most hydrophilic. However, the ideal hydrophilic level of surfaces that ensures the greatest cell adhesion is only to have a contact angle of between 60°–80° [30]. The water absorption ability, on the other hand, has the maximum increase for decellularized ECM with TX100 using the 0.1% concentration and at 4 °C. Apparently, the decellularization increased the capacity of the ECM to absorb water due to larger gaps between collagen fibrils [29]. ECM that are hydrophilic and with higher water absorption values are vital in medical, cosmetics, and therapeutic applications.

It was observed in the thermal analysis of decellularized ECM that the use of 1.0% concentration and 4 °C in the decellularization resulted in an increase in the weight loss of the samples, especially at the second degradation step. In addition, the resulting denaturation temperature measurement of the samples was relatively high compared with the denaturation temperature of human skin of 54.8 °C. Consequently, the developed decellularized ECM could also withstand human temperature experiencing inflammation at around 42 °C [7].

TX100 and SDS are known to be very effective in cell removal and the debris in the decellularized tissue. However, ineffective removal of these detergents in the decellularized ECM poses some undesirable effects on metabolic activity and cytotoxicity that hamper the recellularization process [14,16,24]. The washing method applied in the study was more effective in removing TX100 than SDS. Nevertheless, the washing was still able to reduce the SDS concentrations in decellularized ECM, but the additional investigation must be conducted to determine the metabolic and cytotoxicity effect of these residual concentrations.

## 5. Conclusions

The conversion of milkfish skin to decellularized ECM is a promising endeavor. However, there are still no studies focused on the decellularization of this specific raw material. In the present study, decellularized ECM was developed from milkfish skin using SDS and TX100 as the decellularizing agents. Moreover, the temperature and concentration of the agents were varied, and the developed ECM was characterized. From the results obtained, the decellularization protocols were able to preserve the ECM structure and physical and thermal properties of milkfish skin regardless of the concentration and temperature. Additionally, it was 0.1% sodium dodecyl sulfate (SDS) when used at 4 °C that yielded the most promising results among the different conditions, as shown in Table 2 below.

Future studies could explore more variables to further establish optimized protocols to effectively decellularize milkfish skin while preserving the ECM structure, mechanical, physical, and thermal properties, and biological compatibility and functionality. Additional tests such as GAG quantification, SEM evaluation, and other histological characterizations in supplement with the ones conducted will be vital for future and confirmatory studies.

## Figures and Tables

**Table 1:** Overview of the decellularization conditions.

	Prewashing	Decellularization	Post washing
**Agent**	PBS	0.1% TX100, 1.0% TX100 0.1% SDS, 1.0% SDS	PBS
**Temperature**	Room temperature	4 °C, Room temperature	Room temperature
**Contact Time**	15 min	48 h	15 min × 3

**Table 2:** Summary indicating the best decellularization conditions for the specific tests performed.

Conditions	H&E Staining	DNA Quantification	FTIR	Tensile Strength	Hydrophilicity		Water Absorption	DSC	TGA	Residual Detergent
					(Inner)	(Outer)				
Agent	SDS	SDS	ALL	TX100	SDS	SDS	SDS	TX100	SDS	TX100
Conc	0.1%	1.0%	ALL	0.1%	1.0%	1.0%	0.1%	1.0%	0.1%	0.1%
Temp	4 °C	4 °C	ALL	RT	4 °C	RT	4 °C	4 °C	4 °C	RT

## Data Availability

Not applicable.
